# MiR-590-3p suppresses epithelial-mesenchymal transition in intrahepatic cholangiocarcinoma by inhibiting SIP1 expression

**DOI:** 10.18632/oncotarget.16150

**Published:** 2017-03-13

**Authors:** Chao Zu, Shizhang Liu, Wei Cao, Zongzhi Liu, Hui Qiang, Yong Li, Chong Cheng, Le Ji, Jianhui Li, Jingyuan Li

**Affiliations:** ^1^ Department of Surgical Oncology, Shaanxi Provincial People's Hospital, The Third Affiliated Hospital of Xi’an Jiaotong University, Xi’an, 710068, Shaanxi Province, P.R. China; ^2^ Department of Orthopaedics, Shaanxi Provincial People's Hospital, The Third Affiliated Hospital of Xi’an Jiaotong University, Xi’an, 710068, Shaanxi Province, P.R. China

**Keywords:** intrahepatic cholangiocarcinoma, miR-590-3p, SIP1, metastasis, EMT

## Abstract

The functional roles and clinical significances of miR-590-3p in ICC remain unclear. In the current study, we investigated the expression of miR-590-3p in tissues and sera of ICC by real-time quantitative polymerase chain reaction. We found miR-590-3p was significantly down-regulated in the sera and tissues of ICC patients, especially in those patients with lymph node metastasis or distant metastasis. AUC curves and Cox proportional hazards mode revealed serum miR-590-3p could be novel diagnostic and prognostic biomarker for ICC patients. MiR-590-3p dramatically suppressed epithelial-mesenchymal transition, cell migration, and invasion of ICC cells. *SIP1* was identified as direct and functional target of miR-590-3p in ICC cells by luciferase assays. Finally, we found *SIP1* expression was inversely correlated with miR-590-3p and closely related to diminished survival in ICC patients. These findings reveal functional and mechanistic roles of miR-590-3p and EMT activator *SIP1* in the pathogenesis of ICC.

## INTRODUCTION

Intrahepatic cholangiocarcinoma (ICC) is the second most frequent primary malignant live tumor, and the incidence rate and motility are drastically rising worldwide [1–3]. Surgery is the only potentially curative treatment for ICC patients who have resectable disease. Unfortunately, the clinical outcome of ICC patients are still disappointing, and the median survival time is even less than 24 months [4]. Therefore, it is urgent to explore the molecular mechanism underlying the carcinogenesis, progression, and metastasis of ICC.

The epithelial-to-mesenchymal transition (EMT) is a developmental process in which tightly adherent and polarized epithelial cells are transformed in to loosely organized and nonpolarized mesenchymal cells [5, 6]. The EMT has been identified as pivotal mechanism contributing to the invasiveness and metastatic potential of multiple types of solid cancer, including hepatocellular carcinoma [7, 8], breast cancer [9, 10], prostate cancer [11], colorectal cancer [12], and ICC [13]. MicroRNAs (miRNA) are a group of endogenous small non-coding RNA molecules (approximately 22 nucleotides in length) [14], transcribed from non-protein coding genes or introns. MiRNAs repress mRNA translation or degrade mRNA molecules via binding to their complementary sites in 3'-untranslated region (3'-UTR) [15]. MiRNAs function as tumor suppressor or oncogenes by modulating multiple cellular pathways for proliferation, apoptosis, and invasion [16–18]. More importantly, miRNAs also function as a crucial modulators for EMT by directly targeting transcription factors, such as miR-200/*ZEB1* [19], miR-200/*SIP1* [20]. Though miR-590-3p was reported to inhibit the migration of bladder cancer cells [21], the functional roles and clinical significance of miR-590-3p in ICC remain to be elucidated.

In the current study, we reported miR-590-3p was down-regulated in ICC tissues, sera, and cell lines. Serum miR-590-3p was diagnostic and prognostic biomarker for ICC patients. Moreover, overexpression of miR-590-3p could suppress cell migration, cell invasion, and EMT process by directly targeting *SIP1*. Finally, we found *SIP1* expression was inversely correlated with miR-590-3p and closely related to diminished survival in ICC patients. These results demonstrate a novel role of miR-590-3p in the inhibition of EMT process and highlighting the clinical significance of miR-590-3p in ICC.

## RESULTS

### Expression of miR-590-3p in tissues, sera, and cell lines of ICC

**Figure 1:**
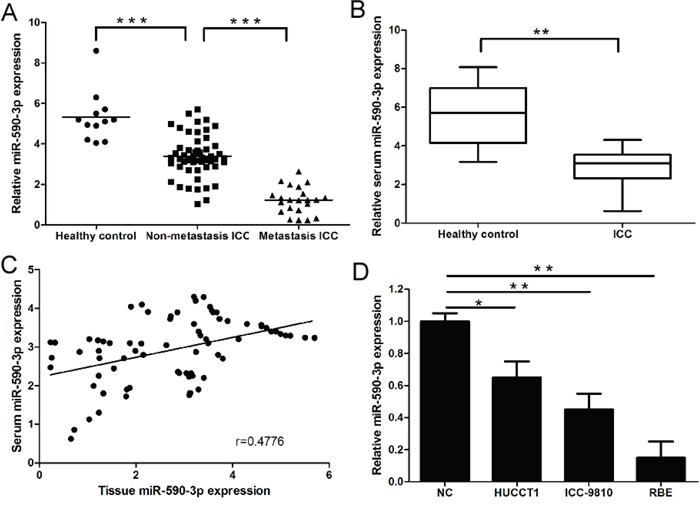
Expression of miR-590-3p in tissues, sera and cell lines of ICC **(A)** Relative expression of miR-590-3p in the tissues of Healthy control, ICC paitents, and ICC patients with lymph node metastasis or distant metastasis; **(B)** Serum levels of miR-590-3p in healthy controls and ICC patients. Boxes represent interquartile range, and the horizontal line across each box indicates median value; **(C)** Spearman's correlation analyses show a significantly inverse correlation between miR-590-3p expression level in tissues and sera of ICC patients; **(D)** Relative expression of miR-590-3p in ICC cell lines; The expression of miR-590-3p was quantified by qRT-PCR and normalized to RNU6B. *P<0.05, **P<0.01,***P<0.001. Serum miR-21 yielded an area under the curve (AUC) value of 0.9081 in distinguishing ICC patients from normal control subjects.

As shown in Figure 1A, miR-590-3p expression was significantly down-regulated in ICC tissues compared to matched normal tissues. Moreover, compared to non-metastatic ICC tissues (n=53), miR-590-3p expression was significantly reduced in metastatic ICC tissues (n=21) (Figure 1A). More importantly, we found serum miR-590-3p expression was also significantly down-regulated in ICC patients compared to healthy controls (Figure 1B). Interestingly, we observed a statistically significantly positive correlation between miR-590-3p expression in tissues and matched serum samples form ICC patients (Figure 1C, r=0.4776, 95%CI: 0.2732 to 0.6408, *P*<0.001; Spearman's correlation analysis), which indicating circulating miR-590-3p expression could accurately reflect concentrations found in ICC tissues. Consistent with these observations, miR-590-3p expression was significantly lower in three ICC cell lines than normal tissues (Figure 1D).

### Diagnostic and prognostic role of serum miR-590-3p in ICC patients

**Figure 2:**
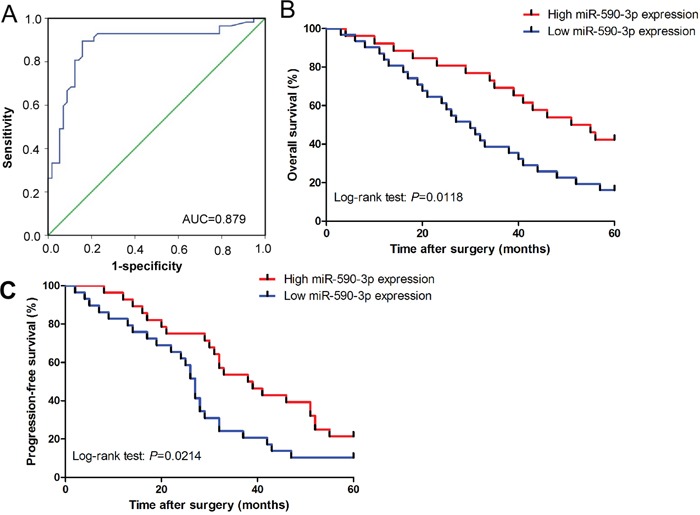
Diagnostic and prognostic role of serum miR-590-3p in ICC patients **(A)** Serum miR-590-3p yielded an area under the curve (AUC) value of 0.879 in distinguishing ICC patients from healthy control subjects; **(B)** and **(C)** Kaplan-Meier plots representing probabilities of progression-free and overall survival in ICC patients according to expression level of serum miR-590-3p.

ROC analysis revealed that serum miR-590-3p levels were robust in discriminating patients with ICC from healthy controls with an AUC value of 0.879 (Figure 2A). Patients with high levels of miR-590-3p in sera and tissues had statistically significantly better overall and progression-free survival (all *P*<0.05; sera: Figure 2B-2C; tissues: Supplementary Figure 1 and 2). Univariate analyses revealed that high serum miR-590-3p expression was good prognosticators for overall and progression-free survival (all *P*<0.05; Table 1-2), whereas TNM stage, lymph node metastasis, distant metastasis were poor prognosticators for overall and progression-free survival in ICC patients. Multivariate analyses demonstrated that high levels of serum miR-590-3p expression was an independent predictor for good overall and progression-free survival in ICC patients (all *P*<0.05; Table 1-2).

**Table 1:** Univariate and Multivariate analysis of clinical parameters in relation to overall survival

Variables	Univariate		Multivariate	
	HR (95%CI)	*P*	HR (95%CI)	*P*
**High miR-590-3p expression**	0.469 (0.219-0.871)	0.026*	0.328(0.311-0.749)	0.008*
**Clinicalstage at diagnosis**	4.589 (1.672-7.309)	0.015*	5.229 (2.183-7.502)	0.007*
**Lymph node metastasis**	2.984 (1.237-4.117)	0.036*	3.109 (1.782-6.181)	0.021*
**Distant metastasis**	2.565 (1.391-5.198)	0.037*	2.994 (1.496-5.115)	0.033*

Abbreviations: CI: confidence interval; HR: hazard ratio

*Significant relation of clinical factors with overall survival.

**Table 2:** Univariate and Multivariate analysis of clinical parameters in relation to progression-free survival

Variables	Univariate		Multivariate	
	HR (95%CI)	*P*	HR (95%CI)	*P*
**High miR-590-3p expression**	0.417 (0.327-0.841)	0.031*	0.514 (0.428-0.891)	0.012*
**Clinicalstage at diagnosis**	5.327 (2.594-9.442)	0.009*	4.978 (1.875-6.892)	0.0011*
**Lymph node metastasis**	2.217 (1.294-4.227)	0.022*	2.574 (1.442-3.908)	0.017*
**Distant metastasis**	3.532 (1.498-5.374)	0.014*	5.117 (1.915-8.162)	0.008*

CI: confidence interval; HR: hazard ratio;

*Significant relation of clinical parameters with progression-free survival

### MiR-590-3p inhibits the migration and invasion of ICC cells

**Figure 3:**
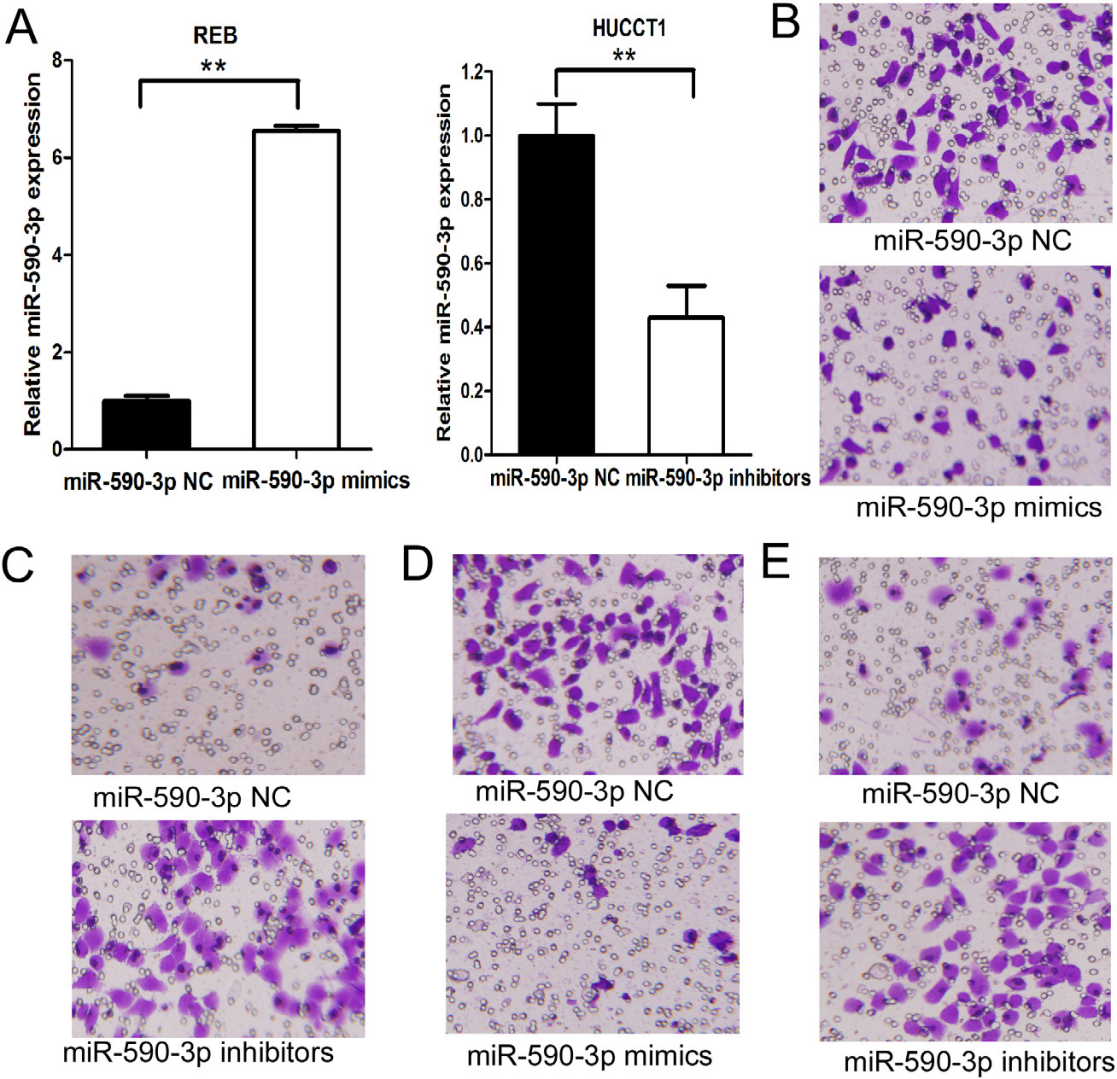
MiR-590-3p inhibits the migration and invasion of ICC cells **(A)** Expression of miR-590-3p in HUCCT1/RBE transfected with miR-590-3p inhibitors/mimics was validated by RT-qPCR. RNU6B was used as an endogenous control; **(B)** Effects of miR-590-3p on cell migration of RBE. **(C)** Effects of miR-590-3p on cell migration of HUCCT1 cells; **(D)** Effects of miR-590-3p on cell invasion of RBE cells. **(E)** Effects of miR-590-3p on cell invasion of HUCCT1 cells.**P<0.01.

We silenced miR-590-3p expression in HUCCT1 cells and established stable-expression of miR-590-3p in RBE cells by transfecting has-miR-590-3p inhibitor and mimics (Figure 3A). As anticipated, up-regulation of miR-590-3p expression significantly suppressed the migration and invasion of RBE cells (Figure 3B and 3D), and miR-590-3p depletion significantly increased the migration and invasion of HUCCT1 cells (Figure 3C and 3E).

### MiR-590-3p suppresses EMT in HUCCT1 cells

**Figure 4:**
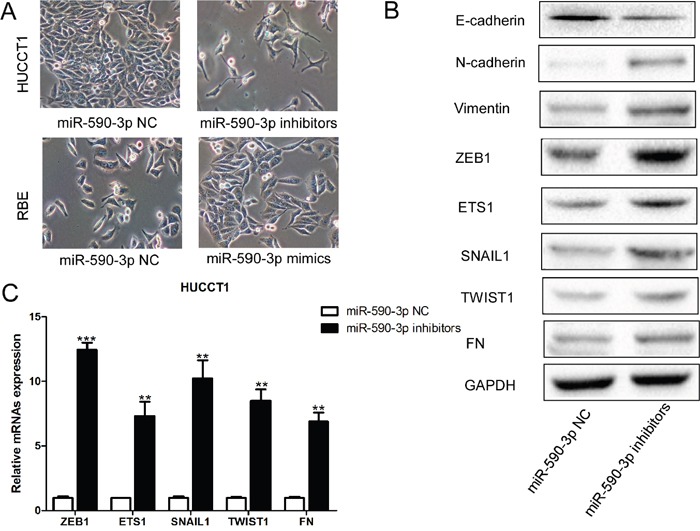
MiR-590-3p suppresses EMT of ICC cells **(A)** Morphological changes of HUCCT1 cells infected with miR-590-3p inhibitors; **(B)** Western blotting analyses of EMT markers in HUCCT1 cells infected with miR-590-3p inhibitors or miR-590-3p NC; **(C)** RT-qPCR analyses of ZEB1, ETS1, SNAIL1, TWIST1, and FN in HUCCT1 cells infected with miR-590-3p inhibitors or miR-590-3p NC.

HUCCT1 cells with miR-590-3p depletion appeared to have lose their tight cell-cell contacts and grew as loosely packed spindle-like fibroblastic cells, which indicating the acquisition of mesenchymal properties; in contrast, RBE cells transfected with miR-590-3p mimics changed from an elongated, fibroblast-like, mesenchymal phenotype to an epithelial cobblestone-like phenotype (Figure 4A). Consistent with the morphological changes of EMT, miR-590-3p depletion in HUCCT1 also resulted in upregulation of mesenchymal protein Vimentin and N-cadherin, and downregulation of epithelial marker E-cadherin (all *P*<0.05; Figure 4B). Moreover, other potent EMT makers were also down-regulated in HUCCT1 cells transfected with miR-590-3p inhibitors, such as ZEB1, ETS1, SNAIL1, TWIST1, and FN (Figure 4B-4C).

### SIP1 is the direct target of miR-590-3p in ICC cells

**Figure 5:**
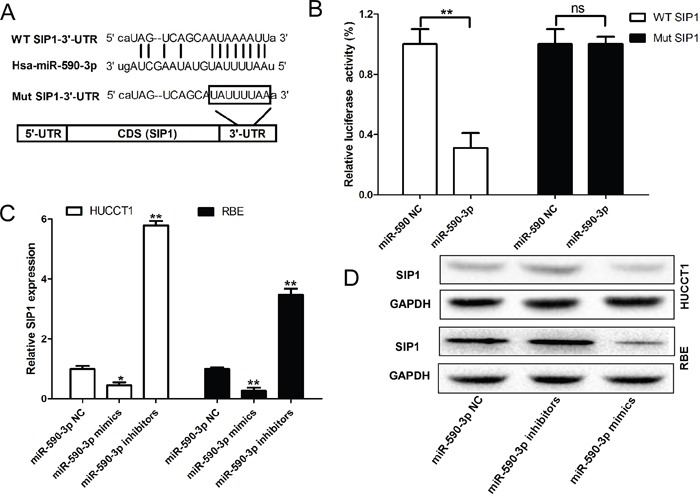
SIP1 are direct target of miR-590-3p **(A)** MiR-590-3p and its putative binding sequence in the 3’-UTR of *SIP1*; diagrammatic representation of the luciferase reporter plasmids with WT and MT *SIP1* 3’-UTR. **(B)** Relative luciferase activity in 293T cells after transfection with WT or MT *SIP1*3’-UTR plasmids co-transfected with miR-21 inhibitors. **(C)** and **(D)** MiR-21 inhibitors/mimics promoted/inhibited the expression level of SIP1 at the mRNA level and protein level in HUCCT1 and RBE cells. Three independent experiments were performed in duplicate. Data are presented as mean ± SD. Two-tailed Student's t test was used. * *P*< 0.05.

We identified Smad-interacting protein 1 (*SIP1*) as a candidate target of miR-590-3p in ICC cells by performed an unbiased computational screen by integrating the results of multiple prediction algorithms [(miRanda, Pic Tar, and Targetscan) (Figure 5A)]. Luciferase reporter assays demonstrated that miR-590-3p significantly repressed activity of reporter vector harboring wild-type (WT) 3'-UTR of *SIP1*, whereas mutations of putative miR-590-3p-binding sites in *SIP1* 3'-UTR regions abrogated the inhibitory effects (Figure 5B). Furthermore, RT-qPCR and western blot analyses showed that mRNA and protein levels of SIP1 were dramatically upregulated in HUCCT1 and RBE cells when miR-590-3p expression was depleted; in contrast, miR-590-3p overexpression substantially decreased SIP1expression in HUCCT1 and REB cells (Figure 5C-5D).

### Repression of SIP1 partially rescues miR-590-3p inhibitors-induced EMT, cell migration and invasion

**Figure 6:**
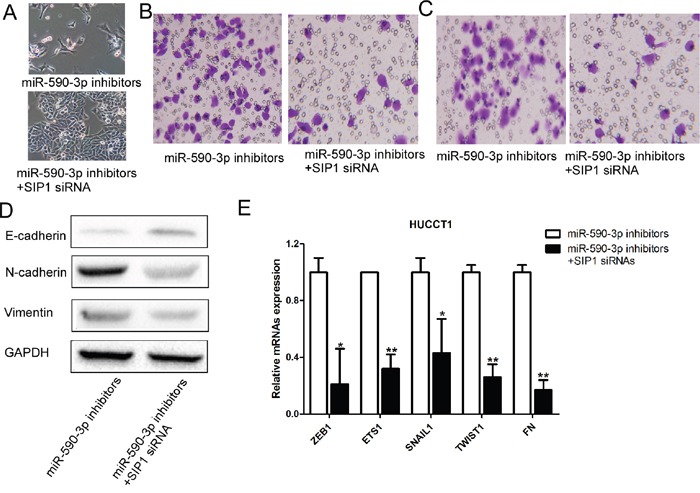
Loss-of-function studies showed that SIP1 siRNA abrogate the of miR-21 inhibitors-induced EMT, cell migration and invasion in ICC *in vitro* **(A)** Morphology assays of HUCCT1 cells infected with miR-590-3p inhibitors or miR-590-3p inhibitors+SIP1 siRNAs; **(B)** Migration assays of HUCCT1 cells infected with miR-590-3p inhibitors or miR-590-3p inhibitors+SIP1 siRNAs; **(C)** Invasion assays of HUCCT1 cells infected with miR-590-3p inhibitors or miR-590-3p inhibitors+SIP1 siRNAs; **(D)** Western blotting analyses of E-cadherin, N-cadhenrin, and Vimentin in HUCCT1 cells infected with miR-590-3p inhibitors or miR-590-3p inhibitors+SIP1 siRNAs; **(E)** RT-qPCR analyses of ZEB1, ETS1, SNAIL1, TWIST1, and FN in HUCCT1 cells infected with miR-590-3p inhibitors or miR-590-3p inhibitors+SIP1 siRNAs.

We performed loss-of-function analyses by silencing SIP1 in HUCCT1 cells transfected miR-590-3p inhibitors. As anticipated, silencing SIP1 could rescue the effects of miR-590-3p inhibitors on HUCCT1 cells, as revealed by cell morphology, cell migration and invasion (Figure 6A-6C). Consistent with these results, upregulation of E-cadherin and N-cadherin and downregulation of Vimentin were observed when SIP1 expression was silenced in HUCCT1 cells transfected with miR-590-3p inhibitors (Figure 6D). Other EMT markers were also found downregulated in HUCCT1 cells transfected with miR-590-3p inhibitors (Figure 6E).

### Clinical significance of SIP1 in ICC

**Figure 7:**
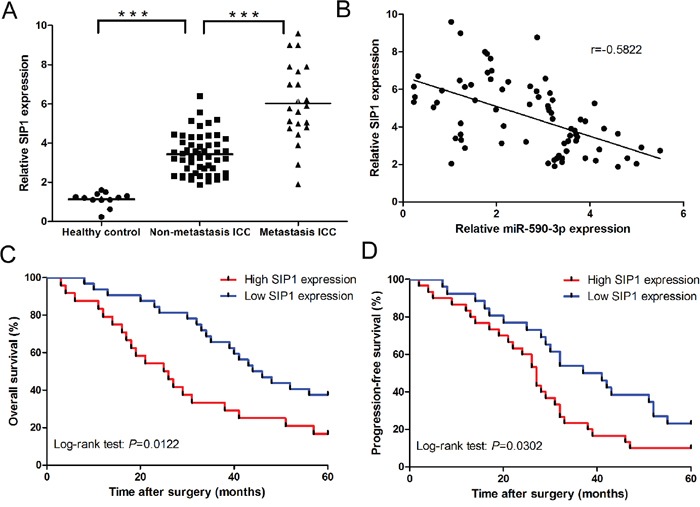
Clinical significance of SIP1 in ICC **(A)** Relative mRNA expression of SIP1 in the tissues of Healthy control, ICC paitents, and ICC patients with lymph node metastasis or distant metastasis. **(B)** Spearman's correlation analyses show a significantly inverse correlation between miR-590-3p expression level and SIP1 mRNA level in ICC tissues; **(C)** and **(D)** Kaplan-Meier plots representing probabilities of progression-free and overall survival in ICC patients according to expression level of SIP1. ***P<0.001.

SIP1 expression was significantly upregulated in ICC tissues compared to healthy control (Figure 7A). Moreover, compared to non-metastatic ICC tissues (n=53), SIP1 expression was significantly upregulated in metastatic ICC tissues (n=21) (Figure 7A). In addition, SIP1 was inversely correlated with miR-590-3p expression in ICC tissues (Figure 7B, r= -0.5822, 95%CI: -0.7189 to -0.4022, *P*<0.001; Spearman's correlation analysis). Kaplan-Meier analyses results indicated patients with high levels of SIP1 had statistically significantly worse overall and progression-free survival (all *P*<0.05; Figure 7C-7D).

## DISCUSSION

MiR-590-3p was reported to simultaneously target four cAMP-dependent protein kinase A (PKA) subunits, including PRKAR1A, PRKAR2B, PRKACB and CFL2. PKA subunits was proved to be important mediators of miR-200c induced inhibition of migration [22]. Thus, miR-590-3p may also play importantly inhibitory role in cell migration and EMT. Furthermore, miR-590-3p has been identified to be a commonly down-regulated miRNA in a wide range of cancer entities [23]. More importantly, it has been confirmed that miR-590-3p overexpression could inhibit cell migration in bladder cancer cell line [21]. Consistent with these previous studies we found miR-590-3p was down-regulated in sera, tissues and cell lines of ICC, especially in metastatic ICC. We additionally demonstrated miR-590-3p could inhibit cell migration, cell invasion, and EMT by targeting oncogene *SIP1* in ICC cell lines. More significantly, serum miR-590-3p was proved to be a diagnostic and independently prognostic indicator for ICC patients. All these results indicated miR-590-3p was a tumor suppressor miRNA and may play a suppressive role in cell migration, cell invasion and EMT of ICC. But we also noticed a recently literature get a contradictory conclusion with our research about the role of miR-590-3p in cancer. In this study, miR-590-3p was reported to be upregulated in hepatocellular carcinoma, and promoted carcinogenesis by inhibiting tumor suppressor gene *PDCD4* and *PTEN* [24]. The discrepancies between our study and this research may reflect the different cancer types and research focus concerned by each study. We focus on the role of miR-590-3p played in cell migration and EMT in ICC cells and the diagnostic and prognostic value of miR-590-3p in ICC patients. But they put their emphasis on the role of miR-590-3p played in cell proliferation in hepatocellular carcinoma cells. The tissue dependent characteristic of miRNA function should also account for the contradict conclusion.

EMT has been considered a critical mechanism involved in cancer progression and metastasis, and more and more investigations have focused the role of EMT in ICC [13, 25–28]. Here, we observed that ectopic expression of miR-590-3p in RBE induced morphological changes from an elongated, fibroblast-like phenotype to an epithelial cobblestone-like phenotype, and inhibited cell migration and cell invasion of ICC cells. Next, we analyzed the expression of invasion suppressor gene E-cadherin and invasion-related genes N-cadherin and Vimentin. Our data showed that miR-590-3p overexpression significantly enhanced E-cadherin expression but decreased the expression of N-cadherin and Vimentin. By contrast, knockdown of miR-590-3p in HUCCT1 cells promoted cell motility and EMT progression.

A series of transcription factors have been reported to promote EMT process in cancer metastasis, including SNAL1, TWIST1, ZEB1, and SIP1 (ZEB2) [29]. SIP1 which is a two-handed E box binding zinc finger transcriptional repressor was initially described as a transcriptional factor collaborating with the TGF-βsignaling pathway by interacting Smad factors [30–32]. SIP1 is frequently upregulated in a variety of human cancers, including pancreatic cancer [33], breast cancer [34], gastric cancer [35], renal caner [36], non-small cell lung cancer [37], hepatocellular carcinoma [38], and ICC [39]. SIP1 was proved to directly bind to and repress E-cadherin expression in cancer cell, thus facilitating the metastasis of cancer cells and inducing EMT [30]. Multiple lines of evidence indicates that upregulation of SIP1 contributes to the invasive and metastatic behavior in multiple types of cancers [34, 38, 40]. A recent published literature showed that high levels of SIP1 protein were significantly associated with cholangiocarcinoma (CCA) metastasis and shorter survival time [39]. Consistent with these previous studies, we found SIP1 was up-regulated in ICC tissues, especially in metastatic ICC. We also found high level of *SIP1* expression was significantly associated with shorter survival time of ICC patients. More importantly, we found *SIP1* was a novel, direct and functional target of miR-590-3p in ICC. These results demonstrate that down-regulation of miR-590-3p will lead to upregulation of *SIP1* in ICC, which promotes metastasis and progression of ICC.

In summary, our results show that miR-590-3p is significantly down-regulated in ICC sera, tissues, and cell lines. We also provide convincing results demonstrating that miR-590-3p inhibit EMT process and suppresses cell migration and cell invasion of ICC. Moreover, *SIP1* is the direct and functional target of miR-590-3p in ICC cells. Lastly, we prove *SIP1* expression is inversely correlated with miR-590-3p expression in ICC tissues and is also the overall and progression-free survival indicator for ICC patients. Enhanced understanding of EMT process that is regulated by miR-590-3p, and the identification of critical targets for individual miRNAs such as *SIP1*, provides novel insight into the mechanism of carcinogenesis and progression in ICC.

## MATERIALS AND METHODS

### Patients and specimens

Both serum- and tissue-based specimen collection and studies were approved by the Research Ethics Committee of Xi’an Jiaotong University. All patients provided written consent and indicated willingness to donate their blood and tissue samples for research. A total of 74 patients were enrolled in this study. 57 patients received curative resection, and 17 patients received palliative resection at Third Affiliated Hospital, Xi’an Jiaotong University (Xi’an, China) from 2007 to 2009. All patients enrolled didn't receive radiotherapy or chemotherapy before operation. All tumors were clinically and histologically diagnosed as intrahepatic cholangiocarcinoma. Inclusion criteria for all cases included: (i) unambiguous histology and absence of mixed tumor types; (ii) absence of any treatment prior to surgery; (iii) age of tissue block less than 7 years. The clinicopahtological characteristics of patients are present as Supplementary Table 1.

### Cell culture

Human ICC cell lines ICC-9810, HUCCT1 and RBE were obtained from the Cell Bank of Chinese Academy of Sciences (Shanghai, China), where they were characterized by mycoplasma detection, DNA-Fingerprinting, isozyme detection and cell vitality detection. They were cultured in RPMI-1640 (Invitrogen, Carlsbad, CA) mediums supplemented with 10% fetal bovine serum (Hyclone, Logan, UT) at 37°C in a humidified atmosphere of 95% air and 5% CO_2_.

### RNA extraction and real-time quantitative polymerase chain reaction (RT-qPCR)

Total RNA was extracted from tissues using TRIzol regent (Invitrogen, Carlsbad, CA) according to the manufacturer's protocol. miRNA were isolated using miRNeasy mini Kit (Qiagen, Valencia, CA, USA). mRNA and miRNA were reversely transcribed to cDNA with the stem-loop reverse transcription primer for mRNA and miRNA detection. Then, mRNA and miRNA expression levels were quantitated using TaqMan miRNA real-time RT-PCR kit (Applied Biosystems) according to the manufacturer's protocol. Data were analyzed with 7500 software v.2.0.1 (Applied Biosystems), with the automatic Ct setting for adapting baseline and threshold for Ct determination. Each sample was examined in triplicate and the amounts of target gene expression (2^-△△^Ct) were normalized using U6 reference. Primers used for RT-qPCR quantification are listed in Supplementary Table 2.

### Ectopic expression vectors of SIP1

For the luciferase reporter plasmid, the 3' UTR of the SIP1, or its mutant variations, was amplified by reverse transcription-quantitative polymerase chain reaction (RT-qPCR), and inserted downstream of the luciferase coding sequence of the pcDNA3.1-luciferase reporter plasmid between the restriction sites BamHI and EcoRI. All the plasmids used in the present study were verified by sequencing.

### Cell migration and cell invasion assays

Cell migration and invasion capacity were measured *in vitro* using transwell migration assays (Millipore, Billerica, MA). The ICC cells were transfected with miR-590-3p mimics, inhibitor or NC for 48h and suspended in RPMI-1640 with 10 g/L BSA at a density of 10^6^ cells/mL. Then, cell suspensions (200μL) were seeded in the upper chamber with apodous membrane coated with (for the transwell invasion assay) or without (for the migration assay) Matrigel (BD Bioscience, San Diego, CA). To attract the cells, 500 μL of RPMI-1640 with 10% serum was added to the bottom chamber. After allowing the cells to migrate for 24 h or to invade for 48 h, the penetrated cells on the filters were fixed in dried methanol and stained in 4 g/L crystal violet. The numbers of migrated or invasive cells were determined from five random fields using a microscope (Olympus) at ×10 magnification.

### Oligonucleotide transfection

MiR-590-3p mimics and inhibitors were chemically synthesized by Shanghai GeneChem (GeneChem, Shanghai, China). Once the cells were 80% confluent, miR-590-3p mimics or inhibitors was transfected into ICC cells with Lipofectamine 2000 (Invitrogen) according to the manufacturer's instructions. Cells were also transfected with scramble oligonucleotide as negative control (NC). The expression level of miR-590-3p in the transfected ICC cells were identified by RT-qPCR.

### Western blotting

Cells were washed in phosphate buffered saline (PBS) three times before proteins were extracted. Then cells were lysed using RIPA buffer, each protein sample (30 mg) was denatured in SDS sample buffer and separated via 10% SDS/PAGE gel. Separated proteins were transferred to polyvinylidene fluoride membranes (Millipore, Billerica, MA, USA) blocked with 5% non-fat milk and incubated overnight with primary antibodies. Blotting was performed with primary antibodies against E-cadherin (ab53226, Abcam, Cambridge, UK), N-candherin (ab18203, Abcam, Cambridge, UK), Vimentin (ab8978, Abcam, Cambridge, UK), ZEB1(ab180905, Abcam, Cambridge, UK), SIP1 ((ab113655, Abcam, Cambridge, UK)), ETS1 (ab10963, Abcam, Cambridge, UK), SNAIL (ab53519, Abcam, Cambridge, UK), TWIST1(ab49254, Abcam, Cambridge, UK), and FN (ab131056, Abcam, Cambridge, UK). Goat anti-rabbit and goat anti-mouse immunoglobulin horseradish peroxidase-linked F(ab)2 fragments (ZAGB-bio, Beijing, China) were used as secondary antibodies. GAPDH (ab181602, Abcam, Cambridge, UK) was used as a loading control.

### Luciferase assays

HEK293T cells were seeded in a 96-well plate at 70% confluence. MiR-590-3p expressiong or control cells were transfected with 30ng of wild type (WT) or mutant (Mut) 3’-UTR of SIP1. The SIP1 3’-UTR was cloned into pMir-Report (Ambion), yielding pMir-Report-SIP1. Mutations were introduced in potential miR-590-3p binding sites using the QuikChange site-directed mutagenesis Kit (Stratagene). Cells were collected 48 hours after transfection, and luciferase assays were measured using a dual luciferase reporter assay system according to the manufacturer's protocol (Promega).

### Statistical analyses

Statistical analyses were performed using IBM SPSS statistical software (version 21.0) (International Business Machines Corporation, Armonk, NY, USA). Mann-Whitney U analyses of variance were used to evaluate statistical differences in serum miRNA expression between unpaired groups. Receiver operating characteristic (ROC) analysis was performed to determine the diagnostic performance of miR-590-3p expression levels in distinguishing patients with ICC form healthy control subjects. Survival curves were estimated using the Kaplan-Meier method, and distributions were evaluated by the long-rank test. Cox proportional hazard models of factors related to survival were used to calculate HRs and identify the factors that affect survival. All *P*-values were determined from 2-sided tests, and statistical significance was based on a *P*-value of 0.05.

## SUPPLEMENTARY MATERIALS FIGURES AND TABLES



## Abbreviations

ICC: Intrahepatic Cholangiocarcinoma
miRNA: MicroRNAs
3’-UTR: 3’-untranslated region
RT-qPCR: Real-time quantitative PCR
IHC: Immunohistochemistry
ROC: Receiver operating characteristic
WT: Wild type
Mut: Mutant
SIP1: Smad-interacting protein 1
AUC: Area under the curve
PKA: cAMP-dependent protein kinase A:
