# Supplementary material for: MiR-590-3p suppresses epithelial-mesenchymal transition in intrahepatic cholangiocarcinoma by inhibiting SIP1 expression
Source: Oncotarget. 2017 Mar 13;8(21):34698–708. doi: 10.18632/oncotarget.16150 (PMC5471004; doi:10.18632/oncotarget.16150)
Supplement: Supplementary file 1 [file oncotarget-08-34698-s001.pdf]

MiR-590-3p suppresses epithelial-mesenchymal transition in intrahepatic cholangiocarcinoma by inhibiting SIP1 expression

SUPPLEMENTARY FIGURES AND TABLES

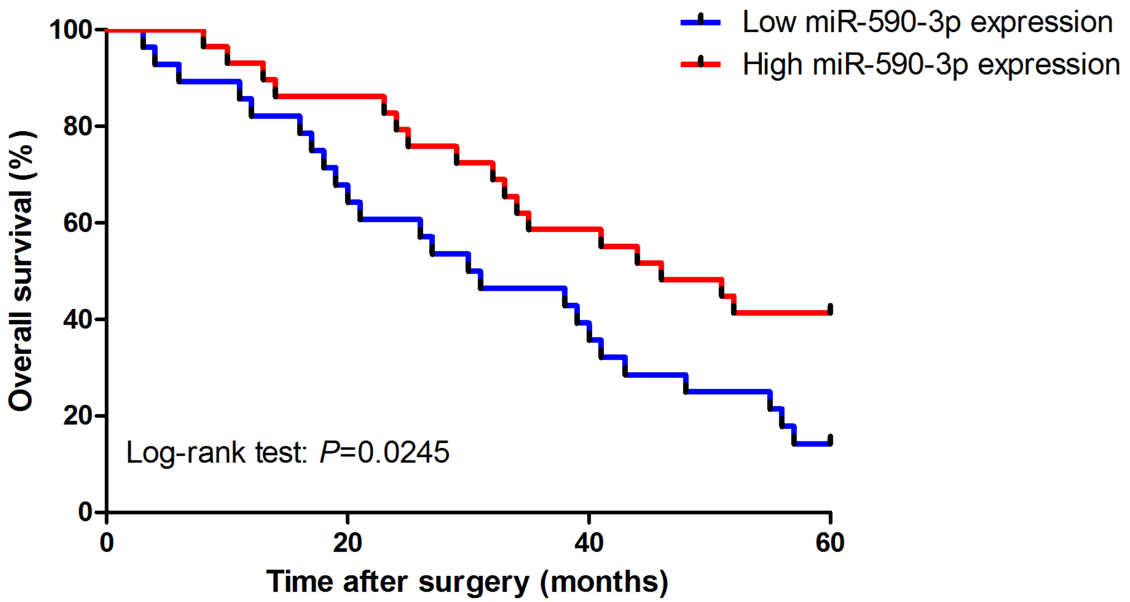

Supplementary Figure 1: Kaplan-Meier plots representing probabilities of overall survival in ICC patients according to expression level of tissue miR-590-3p.

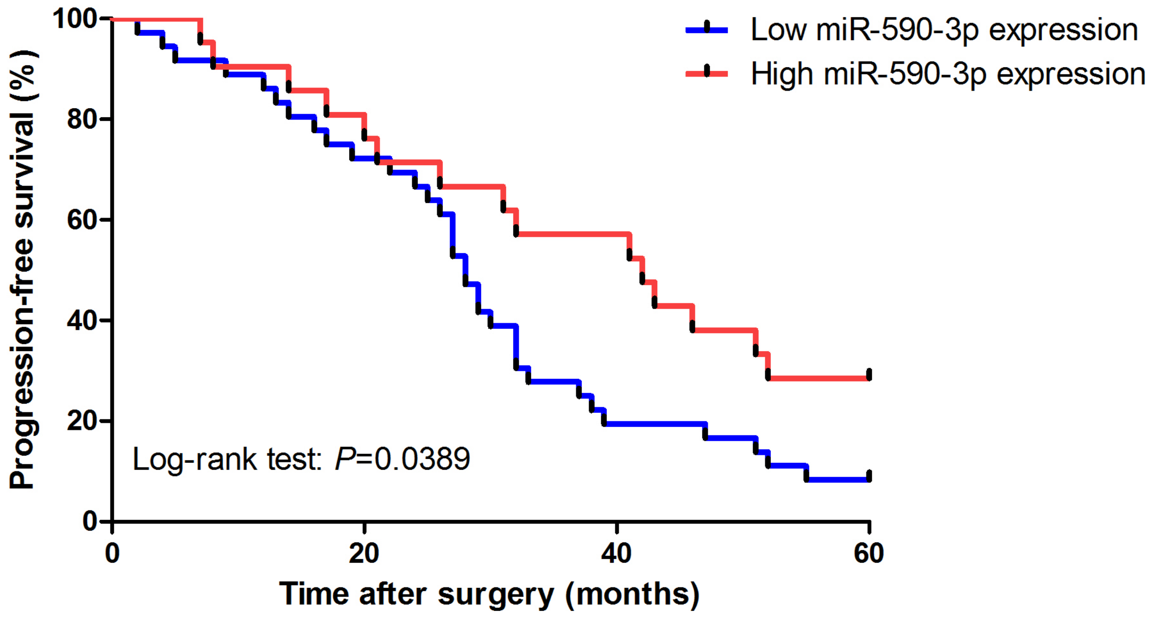

Supplementary Figure 2: Kaplan-Meier plots representing probabilities of progression-free survival in ICC patients according to expression level of tissue miR-590-3p.

Supplementary Table 1: Clinicopathological features of ICC patients

Variables	No.
All patients	74
Age at diagnosis (years)	
≤60	26
>60	48
Gender	
Male	39
Female	35
Clinical stage at diagnosis	
I	5
II	22
III	25
IV	22
Differentiation	
Well	21
Moderate/Poor	53
Tumor origination	
Left	27
Right	38
Bilateral	9

Supplementary Table 2: Primers used for qRT-PCR

Gene name	Forward primer 5'-3'	Reverse primer 5'-3'
**ZEB1**	GACAGTGTTACCAGGGAGGAGCA	TTCAGGTGCCTCAGGAAAAATGA
**SIP1**	CCCTTCTGCGACATAAATACGA	TGTGATTCATGTGCTGCGAGT
**Snail1**	ACAAGCACCAAGAGTCCG	ATGGCAGTGAGAAGGATGTG
**ETS1**	ACAGGGTAAGTGAAGGTTAATTCCA	AGAAAGATGACTACCTTGCTTGACT
**Twist1**	AGCTGAGCAAGATTCAGACCC	GCAGCTTGCCATCTTGGAGT
**FN**	CTGGCCGAAAATACATTGTAAA	CCACAGTCGGGTCAGGAG
**miR-590-3p**	CGGGGGTAATTTTATGTATAAGCTAGT	
**U6**	GTGCTCGCTTCGGCAGCACATAT	
